# Clinical Investigation of Leukocyte DNA Damage in COVID-19 Patients

**DOI:** 10.3390/cimb45020062

**Published:** 2023-01-19

**Authors:** Hasan Doğan, Aslı Kara, Erdem Çankaya, Eda Balkan, Muhammet Ali Gürbüz, Murat Kızılkaya, Merve Aykaç

**Affiliations:** 1 Department of Medical Biology, Faculty of Medicine, Ataturk University, Erzurum 25240, Turkey; 2 Department of Internal Medicine, Erzurum Regional Training and Research Hospital, Health Sciences University, Erzurum 25100, Turkey; 3 Department of Nephrology, Faculty of Medicine, Ataturk University, Erzurum 25240, Turkey; 4 Department of Histology and Embryology, Faculty of Medicine, Ataturk University, Erzurum 25240, Turkey

**Keywords:** COVID-19, comet assay, DNA damage, lymphocyte, lymphopenia, neutrophil/lymphocyte ratio (NLR)

## Abstract

This prospective cross-sectional study aimed to evaluate leukocyte DNA damage in coronavirus disease (COVID-19) patients. In this study, 50 COVID-19-positive patients attending the Erzurum City Hospital Internal Medicine Outpatient Clinic and 42 control group patients were included. DNA damage was detected in living cells through leukocyte isolation in 50 COVID-19-positive patients using the comet assay method. DNA tail/head (olive) moments were evaluated and compared. White blood cells (WBC), red blood cells (RBC), hemoglobin (HGB), neutrophils (NEU), lymphocytes (LYM), eosinophils (EO), monocytes (MONO), basophils (BASO), platelets (PLT), and the neutrophil/lymphocyte ratio (NLR) were analyzed. The RBC, lymphocyte, eosinophil, and monocyte means were significantly higher in the control group (*p* < 0.05), whereas the HGB and neutrophile means were significantly higher in the study group (*p* < 0.05). There were significant negative correlations between COVID-19 and RBC (r = −0.863), LYM (r = −0.542), EO (r = −0.686), and MONO (r = −0.385). Meanwhile, there were significant positive correlations between COVID-19 and HGB (r = 0.863), NEU (r = 0.307), tail moment (r = 0.598), and olive moment (r = 0.582). Both the tail and olive moment mean differences were significantly higher in the study group, with higher ranges (*p* < 0.05). COVID-19 infection caused statistically significant increases in both the tail and olive damage percentage in patients, causing DNA damage. Lastly, the NLR rate was associated with the presence and progression of COVID-19.

## 1. Introduction

COVID-19 is a respiratory disease that has become a global pandemic and has caused numerous deaths. The incubation period of COVID-19 is five days, on average, and the first symptoms appear after 11.5 days in 97.5% of patients [1]. The most common physical symptoms of COVID-19 include fever, cough, dyspnea, myalgia, and fatigue [2,3]. The hospitalization rate of COVID-19-positive patients is 20.7–31.4%, the rate of inpatients in intensive care units is 4.9–11.5%, and the mortality rate is 1.8−3.4% [4]. Clinical sequelae continue after the acute phase of the disease. Fatigue, shortness of breath, cough, headache, loss of taste or smell, and cognitive or mental health disorders are the most common post-acute sequelae of SARS-CoV-2 infection (PASC). Recent studies show that approximately 4–69% of patients are affected by PASC. [5,6].

Studies show that laboratory tests are crucial in terms of recovery rates, severity, malignancy, and treatment follow-up in patients with COVID-19. In patients with COVID-19, lymphopenia, thrombocytopenia, and hypoalbuminemia, and increases in D-dimer, C-Reactive Protein (CRP), creatinine, aminotransferases, cardiac troponins, total bilirubin, erythrocyte sedimentation rate, prothrombin time, and procalcitonin values come to the fore. These clinical features provide information about the diagnosis and prognosis of the disease [7].

Lymphopenia is a condition in which the number of lymphocytes in the blood is low, and there are studies reporting that it is associated with the severity of COVID-19. Lymphopenia was detected in 85% of patients with severe COVID-19 [2,8,9]. Moreover, studies report that individuals who died from COVID-19 also had a significantly lower lymphocyte count than survivors [8]. As a lymphopenia criterion in COVID-19-positive patients, those with a lymphocyte count 1.1 × 10^9^/L were included in four studies [9,10,11,12]. In one study, patients with a lymphocyte count below 1.5 × 10^9^/L were evaluated [13]. Although the number of CD4+ and CD8+ T lymphocytes increased in the early stages of the disease [14], it was found that the number of lymphocytes decreased in the following days [9]. In another study, although natural killer (NK) and cytotoxic T lymphocyte (CTL) cell numbers were significantly reduced in COVID-19 patients, the number of these cells were observed to increase again after recovery [15]. Further, it has been shown that the total number of CD8+ and CD4+ T lymphocytes is considerably reduced in patients with COVID-19 infection, especially in those over 60 years of age and in need of intensive care [16].

The most common severe clinical manifestations in COVID-19 are acute respiratory failure and macrophage activation, both characterized by excessive cytokine storm. In this case, many vital organs, especially the lungs, are affected, which can cause endothelial and vascular dysfunction [17]. Many studies have noted that very high inflammatory cytokine levels in COVID-19 associate cytokine storm with induction of neutrophilia, lymphopenia, and severe illness development [18]. Cytokine storm is defined as an uncontrolled and fatal systemic inflammatory response resulting from the high production of proinflammatory cytokines such as TNF-α, IL-6, IL-1β, and IFN-α. Recent studies indicate that cytokine storm syndrome causes lymphopenia in COVID-19 patients. Cytokines increase lung fibrosis, multi-organ failure, clinically poor prognosis, and mortality in COVID-19 patients. There is a strong link between uncontrolled cytokine production and cytokine storm, morbidity, and mortality in COVID-19 patients [19,20]. Among the cases admitted to intensive care units, various inflammatory cytokines and chemokines, tumor necrosis factor alpha (TNF-α), interferon-ƴ-induced protein 10 (IP-10), monocyte chemoattractant protein 1 (MCP-1), chemokine (CC motif), ligand 3 (CCL-3) and different interleukins (IL-2, IL-6, IL-7, IL-10, etc. were found to be associated with disease severity [2,20,21,22,23,24]. Interestingly, lymphopenia with hypercytokinemia was also found to be prominent in patients with severe SARS-CoV-2 [16,25,26].

Studies have suggested that cytokines may not be the sole cause of lymphopenia and that more than one mechanism may work together to cause lymphopenia [2,13]. SARS-CoV-2 can attack lymphocytes directly or destroy lymphoid organs. Since blood lactic acid levels are elevated in patients with the severe COVID-19 phenotype, lymphopenia may also be induced by such metabolic molecules. Although there are no studies on the induction of apoptosis of NK cells and T cells by SARS-CoV-2, it is thought that viral-based factors may cause lymphopenia by inducing early apoptosis [27]. The factors causing lymphopenia in this context have not been elucidated in the literature, thus, it is unclear which mechanisms lead lymphocytes to apoptosis.

DNA damage results in changes in the basic structure of DNA that disrupt the replication mechanism. DNA damage can be caused by the addition of chemical groups to a DNA base, a chemical change (creating an abnormal nucleotide or nucleotide fragment), or a break in one or both strands of the DNA helix. A study showed that HIV-1 Tat protein causes DNA damage in human peripheral blood B lymphocytes through mitochondrial reactive oxygen species (ROS) production [28]. In another study, it was revealed that latent hepatitis B virus infection with low viremia caused DNA damage, apoptosis, and oxidative stress in peripheral blood lymphocytes. It has also been reported that latent hepatitis C virus triggers mitochondrial oxidative stress in lymphocytes and causes PI3-kinase-mediated DNA damage [29].

DNA damage occurs during signaling, sensing, protein phosphorylation, the complex interaction used by the ubiquitin pathway, and the DNA damage response (DDR) network. In addition, DDR is just one of the many subprograms which makes cell cycle control multifaceted, governed by this network whose central goals are the repair of DNA damage and facilitation of DNA replication. To counter the threats that cause DNA damage, cells have developed various systems to detect DNA damage, realize its presence, and mediate its repair. DNA damage is also caused by reactive oxygen compounds that occur as products of oxidative respiration or through reactions mediated by heavy metals and redox cycle events involving environmental toxins. Reactive oxygen and nitrogen compounds are also produced by macrophages and neutrophils at sites of inflammation and infection. Such chemicals attack DNA, disrupting base pairing, and have effects that disrupt DNA replication and transcription, base loss, or the DNA single-helix structure [30,31]. Therefore, we planned this study to show that the toxins produced by SARS-CoV-2 can cause DNA damage through such interactions.

The COVID-19 pandemic has affected many domains, including daily life, and caused many deaths. Although many studies were conducted on vaccination and prevention during the acute period of the pandemic, those focusing on the effects of the disease have been relatively lacking. Therefore, studies on the short- and long-term effects of COVID-19 on the human body are needed. In this respect, this study can be a foundation for post-COVID-19 DNA and genetic studies. Thus, the study makes an important contribution to the literature.

The comet assay (single-cell gel electrophoresis; SCGE) is a well-known genotoxicity test that is easy to perform. It allows the detection of various DNA damages with high sensitivity in eukaryotic cells exposed to genotoxic agents. In this study, the aim was to evaluate leukocyte DNA damage from COVID-19.

## 2. Materials and Methods

The present study employed a cross-sectional prospective cohort design. A total of 50 COVID-19-positive patients and 42 control group patients were subjects in the study. The subjects were randomly selected from individuals hospitalized with the diagnosis of COVID-19 in the Erzurum City Hospital Internal Medicine Outpatient Clinic.

Patient and control samples were collected in the same period of time. In that period, the COVID-19 Delta variant was seen in our country. Patients who arrived at the hospital within 1–2 days after the onset of symptoms and whose RT-PCR results were positive were included in the study. Peripheral blood samples of the patients were taken before the treatment was started. We included the patients who came to our outpatient clinic and whose collected respiratory samples (oropharyngeal and nasopharyngeal swabs) were confirmed to have COVID-19 via real-time PCR (qRT-PCR) tests in the study group. Swab samples were analyzed on the CFX96 Touch Real-Time PCR Detection system Bio-Rad (CA, USA) with the DS Coronex COVID-19 qPCR test kit (Clydebank, UK) in our hospital. The severity of COVID-19 in the examined patients was determined by the need for hospitalization and treatment for severe illness, that is, individuals who had SpO_2_ < 94% on room air at sea level, a ratio of arterial partial pressure of oxygen to fraction of inspired oxygen (PaO_2_/FiO_2_) <300 mm Hg, a respiratory rate >30 breaths/min, or lung infiltrates >50%. The control group was selected from healthy individuals, aged between 20 and 80, who have not had COVID-19 before, and who do not have any autoimmune or systemic diseases.

While drawing blood for routine tests from the selected patients, 3 mL of blood was collected in an extra heparin tube for the study. In the study, DNA damage was detected in living cells through mononuclear cell isolation in 50 COVID-19-positive patients, including men and women aged 44 and above, and 42 healthy individuals over the age of 42 as the control group. All participants were in included in the study only after their written consent for participation was obtained through the voluntary consent form. All participants gave informed consent, and the local institutional ethics committee approved the study methods.

### 2.1. Total Mononuclear Cell Isolation

Blood samples were transferred to glass tubes (10 × 100 mm) and diluted with phosphate-buffered solution (PBS) at a one-to-one ratio in 15 mL centrifuge tubes, and 3 mL of density gradient medium LymphoprepTM (Density: 1.077 ± 0.001 g/mL, Stemcell Technologies Inc., Oslo, Norway) was added. Tubes were centrifuged at 1134 g for 20 min. The cloud formed by mononuclear cells (PBMC) in the middle of the centrifuged tubes was transferred to a different tube using a thin glass pipette. Thereafter, 10 mL of PBS was added to the tubes, and they were centrifuged at 605 g for 5 min. The supernatant portion of the tubes was discarded, and the above step was repeated. Mononuclear cells obtained after centrifugation were stored in PBS at +4 °C.

### 2.2. DNA Damage Analysis

The collected samples were stored in a −80 °C deep freezer in RPMI-FBS (1/9) solution for an average of 2–3 days before performing the comet assay. DNA damage could be studied in approximately half as many samples as expected.

The neutral comet assay technique of our research was studied in a different laboratory (Atlas Biotechnologies, Ankara, Turkey).

Preparation of Slides: Microscope slides were covered with agarose layer by using 1% normal melting agarose (NMA) (Sigma-Aldrich). NMA (1%) was dissolved in PBS by heating in a microwave without boiling, and then cooling to 65 °C before covering the slides at room temperature. Afterwards, slides were allowed to dry at least overnight at room temperature.

Spread and Denaturation of Cells: Cell suspension was mixed with LMA, and this mixture was spread on an NMA-coated slide. These slides were covered with a coverslip and left on a cold metal plate for 20 min. The coverslips were removed and submerged for 1 h in precooled lysis solution (16.3 mmol/L TritonX-100 (Sigma T8787), 34.08 mmol/L NLauroylsarcosine sodium salt (Sigma L9150), and 8.97 mmol/L DL-Dithiothreitol (DTT) (Vivantis, Shah Alam, Malaysia, PC0705) in 99 mL stock lysis solution). Stock lysis solution consisted of 2.50 mol/L NaCl (Sigma 31434), 99.93 mmol/L EDTA (Vivantis PC0706), and 9.90 mmol/L Trizma (Sigma T1503). Proteinase K (100 mg/mL) (Vivantis PC0712) was added to the lysis solution and mixed gently, after which samples were incubated at 37 °C for 3 h.

Electrophoresis and Fixation: Following the denaturation step, the slides were removed from the lysis solution and placed in an electrophoresis tank containing cold 1X TBE buffer. Electrophoresis was performed in TBE buffer (66 mmol/L Tris-base (Sigma T1503), 67 mmol/L Boric acid (Vivantis PR0607), and 1.5 mmol/L EDTA (Vivantis PC0706) at 1 V/cm for 30 min. The slides were washed in 0.9% NaCl for 90 sec after electrophoresis and dried at room temperature for 1 h. For fixation (10 min), a solution was used that consisted of 0.9 mol/L trichloroacetic acid (Sigma 27242), 0.7 mol/L zinc sulfate heptahydrate (Merck 1.08883), and 68 mmol/L glycerol. The slides were washed three times with deionized water and dried at room temperature. 50 µL (8 µg/mL) ethidium bromide solution (Vivantis PC0707) was dropped on each slide and covered with a coverslip [32].

Imaging: Stained slides were evaluated using a fluorescence microscope (Zeiss, Jena, Germany, Axioscope 5) and Comet IV software (Perceptive Imaging, Liverpool, UK). A total of 50 cells were photographed per patient by taking 10 separate photographs from an average of 10 different fields randomly selected for each sample at ×20 magnification.

Evaluation: Tail/head density in each cell was analyzed according to criteria and the mean % damage values (tail moment and olive moment values) were recorded.

### 2.3. Statistical Methods

Sex distribution was described as frequency and difference analysis was performed by using Fischer’s exact test. Other research variables were described with means and standard deviations. The Kolmogorov–Smirnov test was used for normality of scale parameters. The Mann–Whitney U test was used for non-normally distributed data, and the independent samples *t*-test was utilized for normally distributed data. Since the DNA damage rate of COVID-19 patients is not suitable for normal distribution, non-parametric Spearman’s rho correlation was used for relationship analysis. Lastly, SPSS 17.0 for Windows was used for analysis at a 95% confidence interval with a 0.05 alpha level.

## 3. Results

Sex, age, WBC, basophile, and PLT differences between the study and control groups were not statistically significant (*p* > 0.05). The RBC, lymphocyte, eosinophil, and monocyte means were significantly higher in the control group (*p* < 0.05), whereas the HGB and neutrophil means were significantly higher in the study group (*p* < 0.05) (Table 1).

Both the tail and olive moment means were significantly higher in the study group (*p* < 0.05) (Table 2).

The Spearman’s rho correlation analysis results showed that there were significant negative correlations between COVID-19 and RBC (r = −0.863; *p* < 0.01), LYM (r = −0.542; *p* < 0.01), EO (r = −0.686; *p* < 0.01), and MONO (r = −0.385; *p* < 0.01). Meanwhile, there were significant positive correlations between COVID-19 and HGB (r = 0.863; *p* < 0.01), NEU (r = 0.307; *p* < 0.01), tail moment (r = 0.598; *p* < 0.01), and olive moment (r = 0.582; *p* < 0.01) (Table 3).

The correlation analysis results showed that tail moment was positively correlated with COVID-19 patients (r = 0.598; *p* < 0.01) and HGB (r = 0.573; *p* < 0.01), but negatively correlated with RBC (r = −0.489; *p* < 0.01), LYM (r = −0.288; *p* < 0.01), and EO (r = −0.373; *p* < 0.01). Olive moment was positively correlated with COVID-19 patients (r = 0.582; *p* < 0.01), WBC (r = 0.210; *p* < 0.05), HGB (r = 0.535; *p* < 0.01), and NEU (r = 0.263; *p* > 0.05). Meanwhile, tail moment was negatively correlated with RBC (r = −0.484; *p* < 0.01), LYM (r = −0.281; *p* < 0.01), and EO (r = −0.385; *p* < 0.01) (Table 4).

The results of the Spearman correlation analysis showed that there was a negative correlation between olive moment and ESR with the COVID-19 patients.

**Figure 1:**
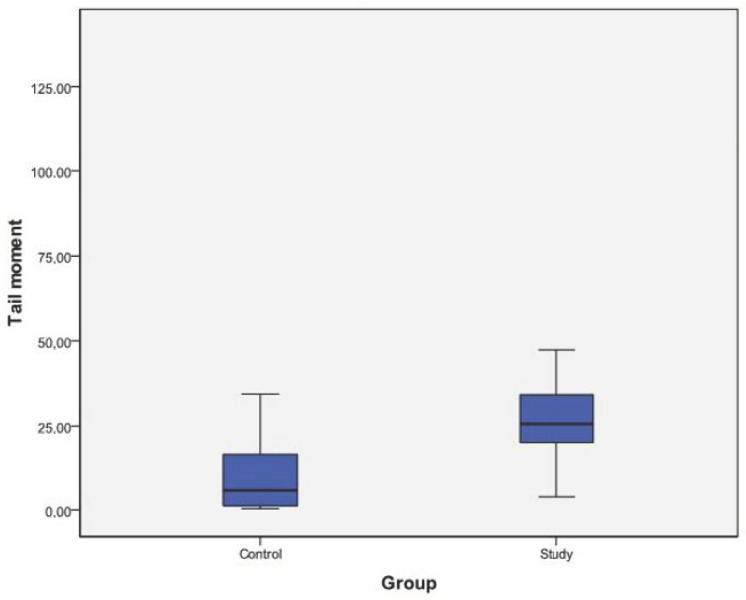
Tail moment ranges and mean differences between the study and control groups.

**Figure 2:**
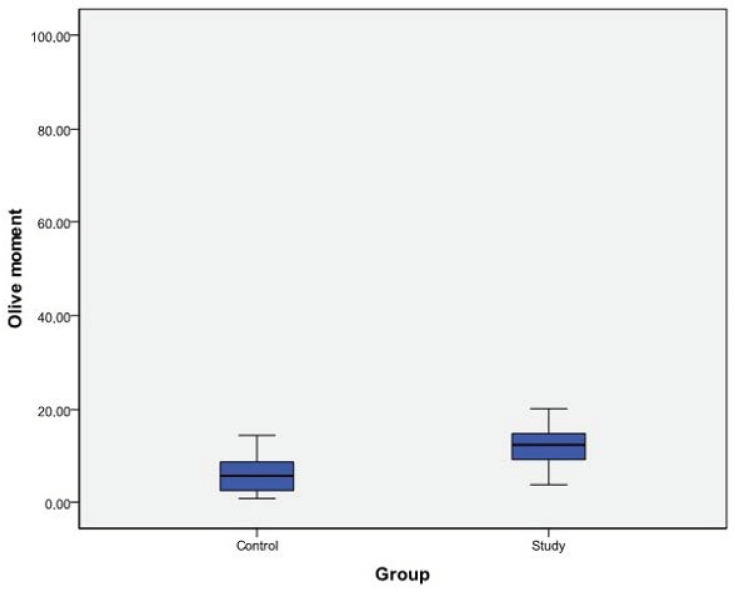
Olive moment ranges and mean differences between the study and control groups.

Both tail and olive moment mean differences were significantly higher in the study group, with higher ranges (Figure 1 and Figure 2).

**Figure 3:**
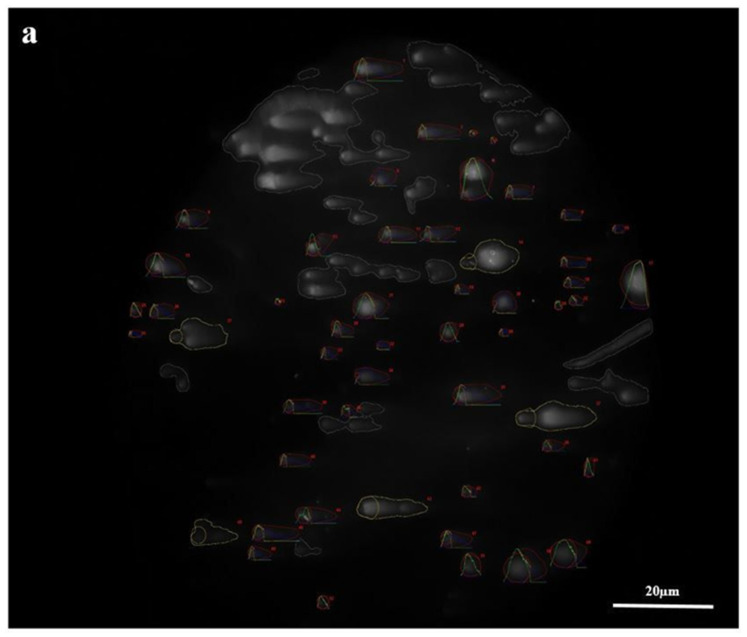

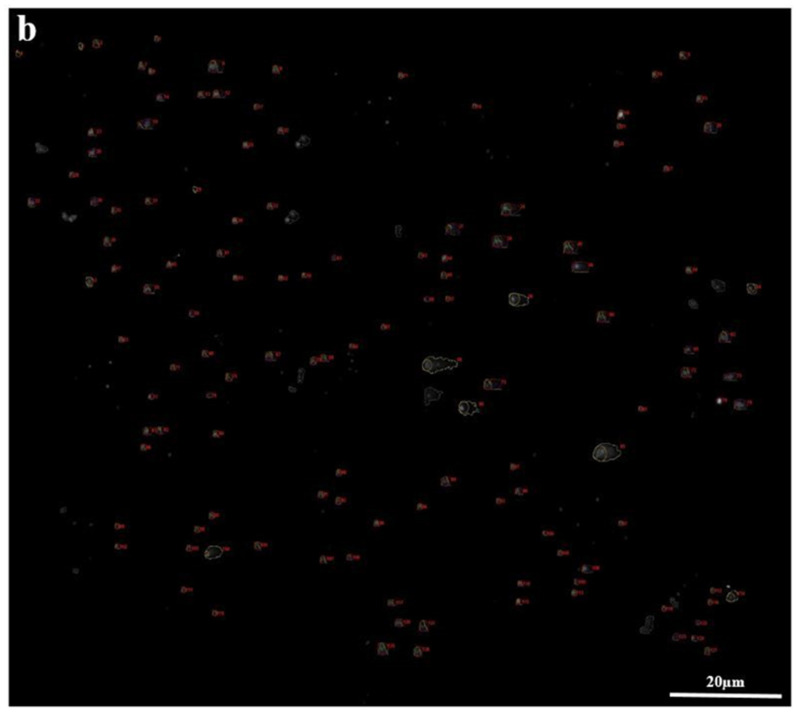
Comet assay distribution of the COVID-19 and control groups (×1000 magnification). The damaged samples of the COVID-19 (**a**) and control (**b**) groups.

The comet assay results showed that tail deformations in the COVID-19 group were definite compared with the control group. To provide an objective comparison, damage samples were selected (Figure 3).

## 4. Discussion

SARS-CoV-2 inflammation can aberrantly activate the DDR, which is involved in immunological differences, DNA damage, micronucleus, telomere shortening, and genome diversity and arrangement. When unrepaired DNA lesions are abundant, they trigger apoptosis or mutagenesis. There is a link between mismatch repair (MMR) and prolonged viral shedding after SARS-CoV-2 infection. The cell repair system is damaged as a result of impaired MMR and oxidative DNA damage caused by the virus [33].

Although COVID-19 has been examined based on different subjects and aspects in relevant literature, hemogram values were included in a large part of the studies. In these studies, statistically significant changes were observed in the hemogram structures of patients after COVID-19, in general [34,35,36,37,38]. In our study, HGB and NEU values were significantly higher in the COVID-19 group, whereas the means of RBC, LYM, EO, MONO, and BASO were statistically significantly higher in the control group. In this respect, it can be stated that there is a concordance between the results obtained in this study and the results reported in the literature.

The number of genetic studies related to COVID-19 is small. These studies suggest that there is a relationship between COVID-19 and genetic structure in general, and that SARS-CoV-2 destroys genetic structure [39,40,41]. However, among these studies, none examined direct DNA damage, hence there are inadequate data to directly compare our research in this regard. According to the data obtained in our study, both tail moment and olive moment averages were statistically significantly higher in the COVID-19 group. When the distributions of tail and olive moment values were examined, the range of variation was higher in the COVID-19 group for both variables. In general, the tail moment change interval was greater than the olive moment variation interval in both groups.

Neutrophil/lymphocyte ratio (NLR) is an indicator of acute inflammation, and many studies are currently examining whether the NLR has diagnostic value for various diseases. In these studies, NLR generally contributes to much more effective results than the diagnostic value or clinical predictive value of neutrophils or lymphocytes alone. In addition, there have been studies in the literature examining the relationship between COVID-19 and NLR. Macchi et al. [42] reported that NLR is an indicator of cytokine storm, and that COVID-19 causes cytokine storm. Therefore, the NLR level may have diagnostic value for COVID-19. In another study, Qin et al. [43] reported that NLR has prognostic value in determining the severity of COVID-19.

In our study, the relationships between COVID-19 and RBC, HGB, NEU, LYM, EO, MONO, tail moment, olive moment, and NLR were statistically significant. Among these correlations, the direction of the relationships between COVID-19 and RBC, LYM, EO, and MONO was negative, whereas the direction of the relationships between COVID-19 and HGB, NEU, tail moment, olive moment, and NLR was positive. While the increase in the NLR level may be an indicator of acute inflammation, the change in blood values can also be expressed by the hematological changes during COVID-19 treatment. However, the fact that tail moment and olive moment changes were significantly related to COVID-19 and the direction of this relationship being positive mean that DNA damage occurs in both the tail and body parts of the DNA of COVID-19 patients.

According to the results obtained in the present study, the relationships between tail moment and COVID-19, RBC, HGB, LYM, EO, and NLR were significant. The relationship between COVID-19, HGB, and NLR and tail moment was positive, while the relationship between RBC, LYM, and EO and tail moment was negative. The relationship between olive moment and COVID-19, WBC, RBC, HGB, NEU, LYM, EO, and NLR was significant. Among these relationships, those between COVID-19, WBC, HGB, NEU, and NLR were positive, while the others were negative. Compared with tail moment, olive moment had a greater effect on hemogram value. On the other hand, COVID-19 affected tail moment more than olive moment. This supports the expected hypothesis proposed in our study. While COVID-19 causes serious DNA damage in patients, the damage is much more evident in the DNA tail region. Meanwhile, considering that the structure of SARS-CoV-2 is mutated and constantly changing, it would be beneficial to evaluate this effect together with new mutations and variations.

Our study has strengths as well as limitations. In the literature review, a study related to COVID-19 and DNA damage such as this was identified to be the first of its kind and, if it published, its impact value would be quite high. However, difficulties in sample collection and isolation of live lymphocytes limited the study. In the planning of the study, we wanted to extract CD4+ and CD8+ cells and show the DNA damage in T and B lymphocytes. However, during COVID-19 inflammation, the lymphocyte count in the patients was very low. We could not make this distinction and we had to analyze the total PBMCs. Nonetheless, highly statistically significant and valuable results were obtained.

The research results showed that COVID-19 infection led to a statistically significant increase in both tail and olive damage percentage in patients, causing DNA damage. In this study, the NLR was associated with the presence and progression of COVID-19. In future studies, conducting research on a larger sample and examining the effect of DNA damage on different types of COVID-19 mutations and different demographic groups would be beneficial.

## Figures and Tables

**Table 1:** Baseline and clinic characteristics of patient groups.

Group	Control (n = 42)	Study (n = 50)	*p* Value
Sex, n (I%)			
Male	31 (73.8)	30 (60.0)	0.120 ^a^
Female	11 (26.2)	20 (40.0)	
Age (Year)	59.29 ± 16.99	62.36 ± 18.18	0.408 ^b^
WBC (10^3^/μL)	9.38 ± 5.02	10.08 ± 4.18	0.116 ^c^
RBC (10^6^/μL)	3.60 ± 0.80	4.56 ± 0.76	<0.05 ^c^
HGB (g/dL)	10.24 ± 2.25	12.91 ± 2.09	<0.05 ^c^
NEU (10^3^/μL)	6.51 ± 4.38	8.45 ± 4.15	<0.05 ^b^
LYM (10^3^/μL)	1.72 ± 0.73	0.99 ± 0.86	<0.05 ^c^
EO (10^3^/μL)	0.29 ± 0.69	0.03 ± 0.07	<0.05 ^c^
MONO (10^3^/μL)	0.83 ± 0.55	0.53 ± 0.29	<0.05 ^b^
BASO (10^3^/μL)	0.04 ± 0.08	0.02 ± 0.02	0.645 ^c^
PLT (10^3^/μL)	226.67 ± 95.54	236.88 ± 79.49	0.577 ^b^
NLR (Ratio)	4.61 ± 3.79	15.62 ± 17.56	<0.05 ^c^

^a^. Fischer’s exact test, ^b^. Independent samples *t*-test, ^c^. Mann–Whitney U test. SD: standard deviation, WBC: white blood cell, RBC: red blood cell, HGB: hemoglobin, NEU: neutrophil, LYM: lymphocyte, EO: eosinophil, MONO: monocyte, BASO: basophil, PLT: platelet, NLR: neutrophil/lymphocyte ratio.

**Table 2:** Tail and olive moment levels of the control and the study group.

Group	Control (n = 42)	Study (n = 50)	*p* Value ^a^
Tail moment	10.30 ± 11.38	30.00 ± 21.21	<0.05
Olive moment	5.99 ± 4.28	13.87 ± 11.64	<0.05

^a^. Mann–Whitney U Test, SD: standard deviation.

**Table 3:** Spearman’s rho correlation analysis results between COVID-19 and significantly different parameters.

Group	r	*p*
RBC (10^6^/μL)	−0.863 **	<0.05
HGB (g/dL)	0.863 **	<0.05
NEU (10^3^/μL)	0.307 **	<0.05
LYM (10^3^/μL)	−0.542 **	<0.05
EO (10^3^/μL)	−0.686 **	<0.05
MONO (10^3^/μL)	−0.385 **	<0.05
Tail moment	0.598 **	<0.05
Olive moment	0.582 **	<0.05
NLR (Ratio)	0.525 **	<0.05

** *p* < 0.01, RBC: red blood cell, HGB: hemoglobin, NEU: neutrophil, LYM: lymphocyte, EO: eosinophil, MONO: monocyte, NLR: neutrophil/lymphocyte ratio.

**Table 4:** Correlation between baseline characteristics, tail moment, and olive moment of COVID-19 patients.

	Tail Moment	Olive Moment
COVID-19	0.598 **	0.582 **
Sex	0.058	0.107
Age (Year)	0.018	0.057
WBC (10^3^/μL)	0.147	0.210 *
RBC (10^6^/μL)	−0.489 **	−0.484 **
HGB (g/dL)	0.573 **	0.535 **
NEU (10^3^/μL)	0.201	0.263 *
LYM (10^3^/μL)	−0.288 **	−0.281 **
EO (10^3^/μL)	−0.373 **	−0.385 **
MONO (10^3^/μL)	−0.154	−0.124
BASO (10^3^/μL)	0.062	0.075
PLT (10^3^/μL)	−0.054	−0.075
NLR (Ratio)	0.297 **	0.330 **

* *p* < 0.05 ** *p* < 0.01 WBC: white blood cell, RBC: red blood cell, HGB: hemoglobin, NEU: neutrophil, LYM: lymphocyte, EO: eosinophil, MONO: monocyte, BASO: basophil, PLT: platelet, NLR: neutrophil/lymphocyte ratio.

## Data Availability

The data presented in this study are available on request from the corresponding author.
